# A Cell Culture Model of BK Polyomavirus Persistence, Genome Recombination, and Reactivation

**DOI:** 10.1128/mBio.02356-21

**Published:** 2021-09-02

**Authors:** Linbo Zhao, Michael J. Imperiale

**Affiliations:** a Department of Microbiology and Immunology, University of Michigan, Ann Arbor, Michigan, USA; b Comprehensive Cancer Center, University of Michigan, Ann Arbor, Michigan, USA; University of North Carolina, Chapel Hill

**Keywords:** BKPyV, persistent infection, polyomavirus, recombination

## Abstract

BK polyomavirus (BKPyV) is a small nonenveloped DNA virus that establishes a ubiquitous, asymptomatic, and lifelong persistent infection in at least 80% of the world's population. In some immunosuppressed transplant recipients, BKPyV reactivation causes polyomavirus-associated nephropathy and hemorrhagic cystitis. We report a novel *in vitro* model of BKPyV persistence and reactivation using a BKPyV natural host cell line. In this system, viral genome loads remain constant for various times after establishment of persistent infection, during which BKPyV undergoes extensive random genome recombination. Certain recombination events result in viral DNA amplification and protein expression, resulting in production of viruses with enhanced replication ability.

## INTRODUCTION

Polyomaviruses are a group of small nonenveloped icosahedral DNA viruses about 45 nm in diameter (1). The first polyomavirus was identified in 1953 as a filterable agent that causes salivary gland carcinomas in mice, and two human polyomaviruses were subsequently identified in 1971: BK polyomavirus (BKPyV) was isolated from a kidney transplant patient with the initials B.K. who was hospitalized for a ureteric obstruction (2) and JC polyomavirus was cultivated from a progressive multifocal leukoencephalopathy patient with the initials J.C. (3). Epidemiology studies have shown that BKPyV infection is ubiquitous among the world population, with more than 80% of the population serologically positive for BKPyV (4). After initial exposure, BKPyV establishes a persistent asymptomatic infection in the urinary tract with periodic shedding of infectious virus particles into the urine of healthy individuals (5). However, in some immunosuppressed patients, BKPyV reactivates and replicates to high levels, causing polyomavirus-associated nephropathy in renal transplant recipients and hemorrhagic cystitis in allogeneic hematopoietic cell transplant recipients (6). The mechanisms underlying BKPyV persistence and reactivation are not understood.

**FIG 1:**
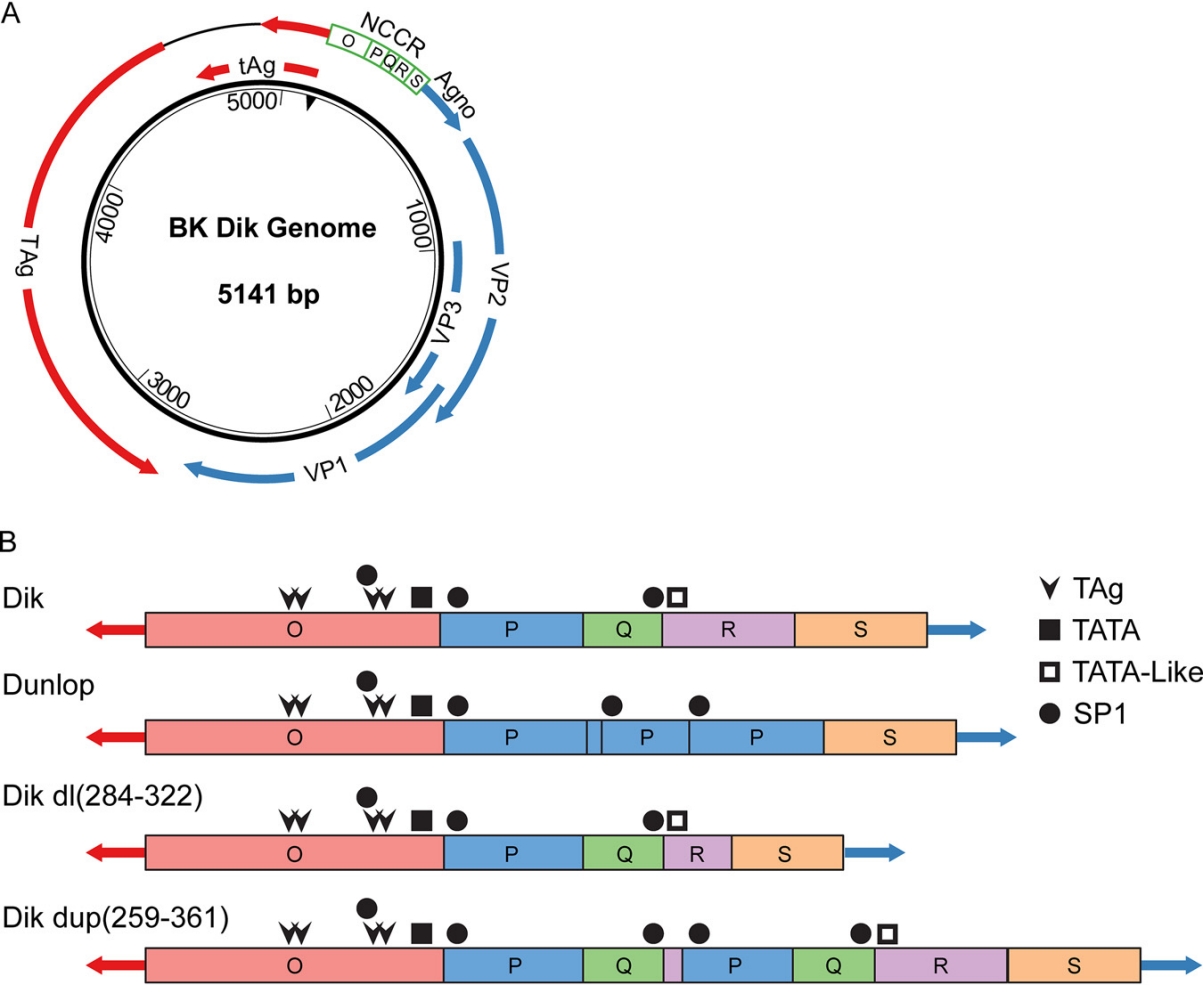
Diagrams of the BKPyV genome and NCCRs. (A) Schematic diagram of the BKPyV genome. Arrows indicate open reading frames. See text for explanation of nucleotide numbering. Color coding of the viral genome is consistent throughout all the figures. (B) The sequence blocks of the archetype (Dik), Dunlop variant, and recombinant NCCRs described in this paper are shown. The binding sites for factors discussed in the text (TAg, Sp1), as well as the putative TATA and TATA-like elements, are indicated.

BKPyV has a circular double-stranded DNA genome about 5 kb in size, which is divided into three functional regions: early region, late region, and noncoding control region (NCCR) (Fig. 1A). The NCCR contains the origin of DNA replication along with *cis*-acting elements that regulate the bidirectional transcription of both early and late genes from opposite strands of the genome (7). There are two genetic forms of BKPyV, which are distinguished by the structure of the NCCR: archetype virus and rearranged variants. Archetype BKPyV is commonly isolated from healthy individuals, and it is regarded as the persistent and transmissible type of virus (8). However, this virus replicates poorly if at all in cell culture (7, 9). The NCCR of archetype virus is arbitrarily divided into five sequence blocks termed O, P, Q, R, and S, while rearranged variants usually have duplications and/or deletions of these blocks (Fig. 1B), and it has been shown that the NCCR is the major determinant of replication ability (7, 10, 11). Rearranged variants are commonly isolated from patients with BKPyV disease and can replicate robustly *in vitro* (12), including in cultured primary human renal proximal tubule epithelial (RPTE) cells (13). Because of a lack of a suitable animal or cell culture model, less is known about infection with archetype virus and the evolutionary processes that lead from archetype virus to rearranged variants. While it is thought that rearranged NCCRs are generated by recombination, direct evidence for this process remains elusive (14, 15).

In this study, we established an *in vitro* model of archetype BKPyV persistence using a human telomerase reverse transcriptase-expressing human RPTE cell line (RPTE-hTERT) (16). This model mimics the immunosuppressed environment in transplant patients in that no adaptive immune system is present. We find that archetype BKPyV can persist in RPTE-hTERT cells for up to 100 days before reactivation and genome amplification occur. We show that amplification is associated with robust recombination of the viral genome. We carefully mapped the recombination events in BKPyV genomes harvested from different time points during the persistence stage and reactivation. Our next-generation and single-molecule sequencing results indicate that the BKPyV genome continuously evolves with random recombination during persistent infection. Eventually, these recombination events lead to rearrangements in the NCCR that allow increased BKPyV genome replication, gene expression, and production of progeny virus that has enhanced replication ability when used to infect naive cells. Analysis of the recombination junctions indicates that recombination primarily appears to occur by nonhomologous end joining (NHEJ). This model will be useful for future studies of the viral and host factors that mediate persistence and recombination.

## RESULTS

### Archetype BKPyV virus establishes a persistent infection and then reactivates in RPTE-hTERT cells.

While an *in vitro* acute BKPyV lytic infection model of RPTE cells with which to study rearranged variants exists (13), primary cells cannot be easily adapted to a persistent infection model for archetype virus due to their limited capability to replicate in culture. As our previous studies showed that BKPyV behaves differently in its natural host cells (RPTE) and nonphysiological cell lines such as CV-1 and Vero cells (17, 18), we wished to establish a persistence model by minimally modifying the natural host cells. To overcome the growth limit of primary RPTE cells, we developed a human telomerase reverse transcriptase-expressing RPTE cell line (RPTE-hTERT), which is immortalized but behaves identically to primary cells with respect to lytic infection by rearranged variants (16).

**FIG 2:**
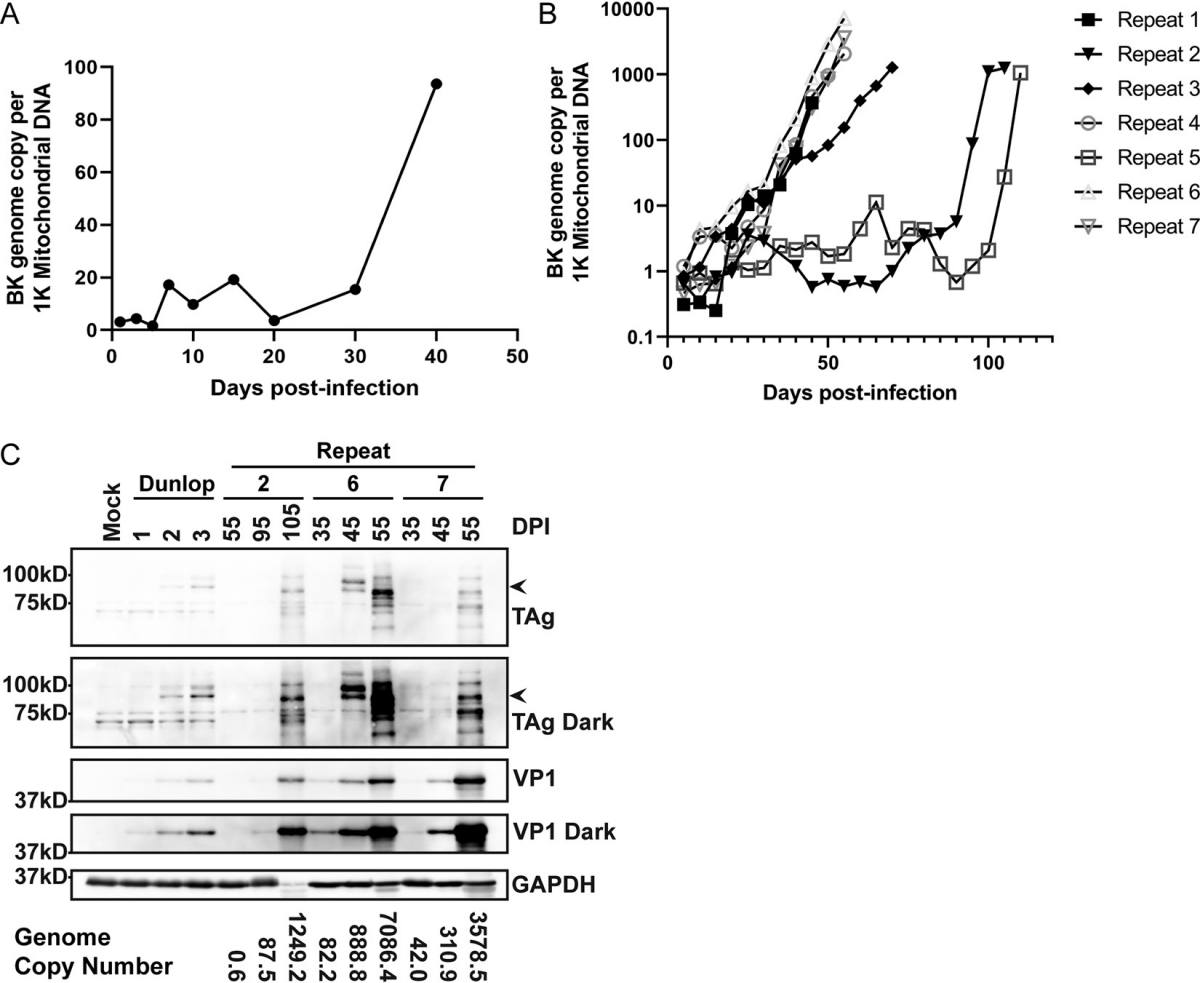
BKPyV genome copy number and viral protein expression during persistence and reactivation. RPTE-hTERT cells were infected at an MOI of 5,000 genomes/cell, and samples were harvested as described in the text. (A) BKPyV genome copy numbers at the indicated time points postinfection were determined by qPCR and normalized to mitochondrial DNA copy number. (B) BKPyV genome copy numbers for the seven repeats were determined as in panel A every 5 days postinfection. (C) Protein samples from persistent infection samples and infection with a rearranged variant of BKPyV (Dunlop) were analyzed for viral early (TAg) and late (VP1) protein expression, indicated by the arrowheads. The DNA copy number for each corresponding sample is indicated at the bottom of the blot. DPI, days postinfection; GAPDH, glyceraldhyde-3-phosphate dehydrogenase.

To test if RPTE-hTERT cells can be used as a persistence model for BKPyV, we infected the cells with archetype virus. Cells were passaged, and low-molecular-weight DNA was harvested at various time points postinfection until obvious cytopathic effect (CPE) resulted in an inability to passage the cells further. Viral genome copy numbers were measured by quantitative PCR (qPCR) and normalized to mitochondrial DNA copy number, as a surrogate for cell number (mitochondrial DNA is extracted with low-molecular-weight viral DNA). This pilot experiment showed that BKPyV persisted at a low genome copy number in RPTE-hTERT cells for 30 days before genome replication and reactivation occurred (Fig. 2A). Cells infected with a rearranged variant, however, died in less than a week. To confirm and expand upon this result, we performed seven additional repeats, passaging the cells and harvesting DNA and protein every 5 days. Our overall results show that BKPyV establishes a persistent infection in RPTE-hTERT cells and maintains a low copy number for 20 to 100 days before reactivation and genome amplification occur (Fig. 2B). An average kidney cell has approximately 1,000 mitochondria (19); therefore, there are fewer than 10 archetype genomes per cell during persistence. Because the cells were passaged at a 1:2 ratio each time and yet the BKPyV genome copy level remained stable for up to 100 days, we conclude that BKPyV is replicating at a low level in RPTE-hTERT cells in order to keep up with cell division. After reactivation, the BKPyV genome copy number increased exponentially until CPE and cell death were observed in each experiment.

To examine viral protein expression during persistence and reactivation, samples collected at representative time points were examined by Western blotting for viral early (large T antigen [TAg]) and late (VP1) proteins (Fig. 2C). Proteins from infection with a rearranged variant of BKPyV (Dunlop) at 1 to 3 days postinfection (DPI) were included as a positive control. The results showed that the level of viral protein expression correlates with the genome copy number. TAg and VP1 were barely detectable at 55 days in repeat 2 and at 35 days in repeats 6 and 7. Both TAg and VP1 expression increased as genome copy number increased, indicating progression through the viral life cycle. We also observed variation in TAg protein sizes as the infections progressed, and analysis of the sequences shows examples of deletions that might explain some of the variation (see Fig. S1 in the supplemental material). These results suggest that BKPyV establishes a persistent infection and eventually reactivates in this *in vitro* model.

10.1128/mBio.02356-21.1 FIG S1 Examples of recombination within the TAg open reading frame (ORF). Representative examples of deletions that might explain the size variation in Fig. 2C are shown, along with locations of functional domains (J domain, LXCXE Rb binding domain, origin binding domain) for reference. Download FIG S1, TIF file, 1.9 MB. Copyright © 2021 Zhao and Imperiale. 2021 Zhao and Imperiale. https://creativecommons.org/licenses/by/4.0/ This content is distributed under the terms of the Creative Commons Attribution 4.0 International license.

Given the variability in the length of the persistent phase of the infection, we wondered whether we might be able to obtain similar data from an infection of primary cells if robust replication began early. We therefore infected primary RPTE cells in the same manner. In two experiments, we obtained a short-enough period of persistence to allow for significant replication before the life span limit of the cells was reached (Fig. S2). These data indicate that the parameters of our system are not an artifact of cell immortalization with hTERT.

10.1128/mBio.02356-21.2 FIG S2 BKPyV genome copy number during persistence and reactivation in RPTE cells. BKPyV genome copy numbers at the indicated time points postinfection were determined by qPCR and normalized to mitochondrial DNA copy number as in Fig. 2. Download FIG S2, TIF file, 2.2 MB. Copyright © 2021 Zhao and Imperiale. 2021 Zhao and Imperiale. https://creativecommons.org/licenses/by/4.0/ This content is distributed under the terms of the Creative Commons Attribution 4.0 International license.

### BKPyV genomic recombinants begin to accumulate during persistence.

It is generally thought that rearranged variants of BKPyV are derived by recombination of archetype genomes during persistent infection, followed by selection for viruses with enhanced replication ability (14), but this has not been proven experimentally. We therefore sequenced BKPyV genomes over the course of our experiment and examined if recombination was occurring during persistent infection. We prepared DNA libraries directly from low-molecular-weight DNA with a transposon-based library preparation kit to minimize recombination artifacts that could be generated during the DNA library preparation (20). We also started with a maximum amount of the DNA template to minimize the number of PCR cycles required to barcode the library, such that we were able to prepare all libraries with only five PCR cycles. Low-molecular-weight DNA from uninfected cells and a plasmid containing the archetype BKPyV genome were included as controls and to validate the recombination identification pipeline. After quality checking and pooling the barcoded libraries, DNA sequences were acquired by reading 250 bp from both ends of each DNA fragment. Three hundred thousand to 600,000 reads were successfully obtained from each sample. All reads without at least one stretch of 25 bp that matched the archetype viral genome were discarded. The remaining reads were aligned against an archetype genome template using the Basic Local Alignment Search Tool (BLAST), and analysis was performed as described in Materials and Methods to identify recombination events. Out of more than 470,000 reads from each negative control, we detected only 3 apparent recombination events in our uninfected control and 247 false recombination events (∼0.05% of the total reads) in our plasmid control. These false-positive events are probably due to pooled sequencing result parsing errors and low levels of artifactual recombination introduced by PCR during the library preparation step in the plasmid sample. The overall low false-positive rate suggests that our library preparation protocol and recombination detection assay were robust and not prone to artifacts. In the experimental samples, we detected a very large increase over time in the number of reads containing recombined segments of the viral genome. During the exponential replication stage, 6.7% to 14.1% of the total reads contained recombined viral segments.

**FIG 3:**
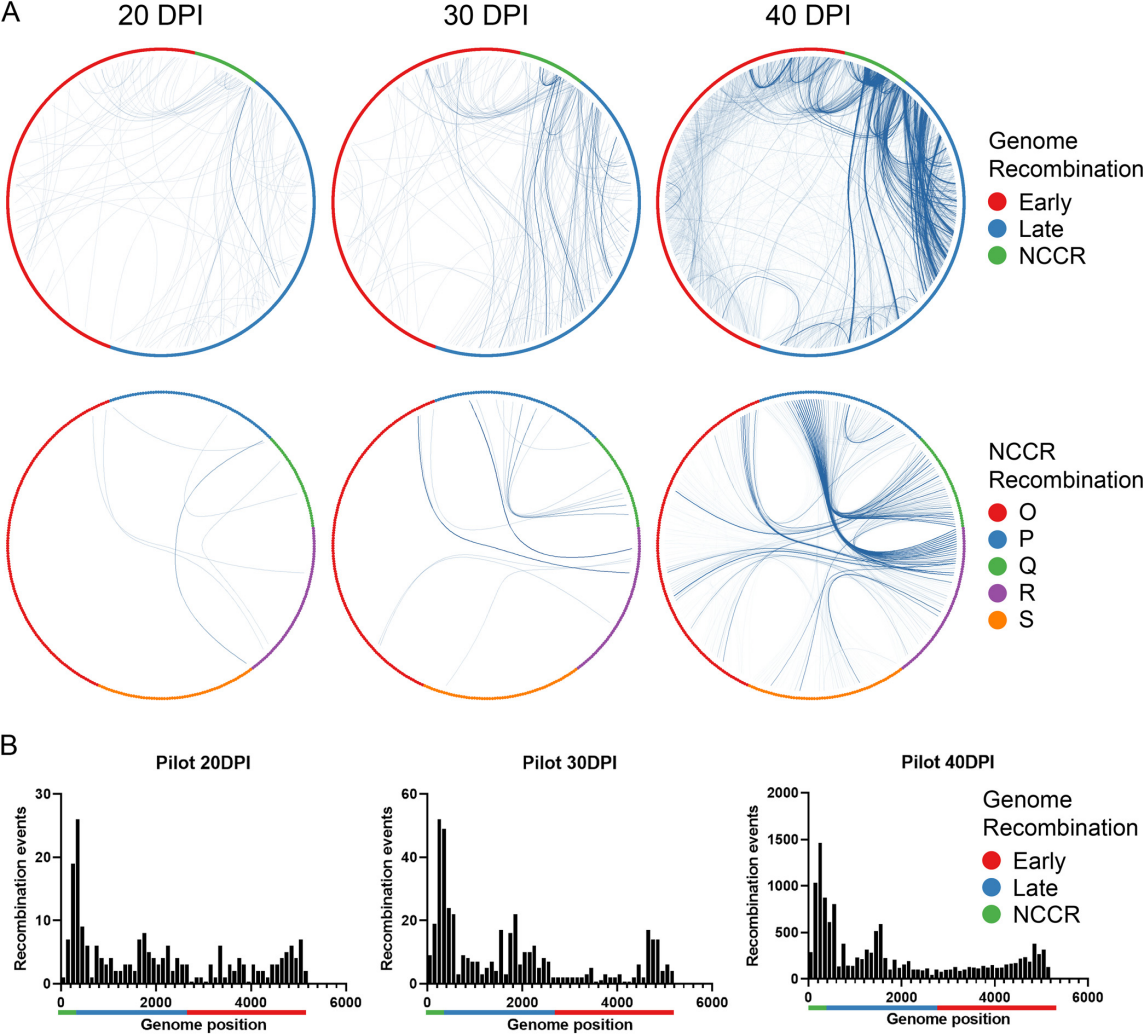
BKPyV genome recombination during persistence and reactivation. (A) BKPyV recombination junctions within the whole genome (row 1) or NCCR (row 2) at the indicated time points from the pilot experiment are depicted as circular diagrams in which the junctions are linked with lines. The 40-DPI diagrams were generated with long-read sequencing. (B) Distribution of unique recombination events. The distribution of recombination events at representative time points is illustrated as a histogram, with each bar reflecting 100 bp. Note the different *y* axis scales. *x* axis legend: green bar, NCCR; red bar, early region; blue bar, late region.

To facilitate the analysis, we took the Dik genomic sequence and arbitrarily reassigned the number 1 to nucleotide 5034 in the GenBank sequence, which is the first nucleotide in the NCCR (Fig. 1A). Thus, the sequence we used starts at the early region junction of the NCCR and ends with the TAg start codon on the opposite strand. The results from the original experiment and three representative experiments from the seven additional repeats are depicted as circular diagrams in which the recombination junctions are linked with lines (Fig. 3A, row 1; see also Fig. S3). The intensity of each line reflects the number of reads corresponding to that junction. The data show that there is extensive recombination in the BKPyV genome and that new recombination events continue to arise as infection progresses.

10.1128/mBio.02356-21.3 FIG S3 BKPyV genome recombination during persistence and reactivation. Recombination junctions at representative time points from three of the repeats are depicted as lines joining positions on the circular genome as in Fig. 3A. The intensity of each line reflects the number of reads corresponding to each junction. Download FIG S3, TIF file, 2.5 MB. Copyright © 2021 Zhao and Imperiale. 2021 Zhao and Imperiale. https://creativecommons.org/licenses/by/4.0/ This content is distributed under the terms of the Creative Commons Attribution 4.0 International license.

To view recombination events within the NCCR in greater detail, we also graphed them in the circular format (Fig. 3A, row 2; Fig. S4). These diagrams show that recombination in the P and Q blocks appears to be enriched, suggesting that P and Q block recombination is more likely to benefit viral genome replication, and which is consistent with previous analyses of NCCR function (21). In addition, we graphed the distribution of recombination events as histograms as a more quantitative means of indicating where recombination occurred over time. The histograms show that recombination is fairly evenly distributed in the viral genome at the earlier time points, but at the later time points, genomes with NCCR recombination become more abundant (Fig. 3B; also Fig. S5 and S6). These results suggest that the BKPyV genome accumulates a significant amount of recombination events and evolves rapidly.

10.1128/mBio.02356-21.4 FIG S4 BKPyV recombination within the NCCR during persistence and reactivation. Recombination junctions within the NCCR at representative time points are depicted as in Fig. S3 but showing just the NCCR. The intensity of each line reflects the number of reads corresponding to each junction. Download FIG S4, TIF file, 2.5 MB. Copyright © 2021 Zhao and Imperiale. 2021 Zhao and Imperiale. https://creativecommons.org/licenses/by/4.0/ This content is distributed under the terms of the Creative Commons Attribution 4.0 International license.

10.1128/mBio.02356-21.5 FIG S5 Distribution of unique recombination events. Genome recombination events at representative time points from the pilot and three repeats are illustrated as histograms with each bar representing 100 bp. Green bar, NCCR; red bar, early region; blue bar, late region. Download FIG S5, TIF file, 2.1 MB. Copyright © 2021 Zhao and Imperiale. 2021 Zhao and Imperiale. https://creativecommons.org/licenses/by/4.0/ This content is distributed under the terms of the Creative Commons Attribution 4.0 International license.

10.1128/mBio.02356-21.6 FIG S6 Average copy number of each unique recombination junction. The average copy number was calculated as total recombination read counts/unique recombination counts (deep sequencing reads per unique recombination junction) in each 380-bp segment of the genome. Results from pilot experiment at 40 DPI, repeat 2 at 105 DPI, repeat 6 at 55 DPI, and repeat 7 at 55 DPI are plotted together. Green bar, NCCR; red bar, early region; blue bar, late region. Download FIG S6, TIF file, 0.8 MB. Copyright © 2021 Zhao and Imperiale. 2021 Zhao and Imperiale. https://creativecommons.org/licenses/by/4.0/ This content is distributed under the terms of the Creative Commons Attribution 4.0 International license.

### BKPyV recombination enhances viral replication.

**FIG 4:**
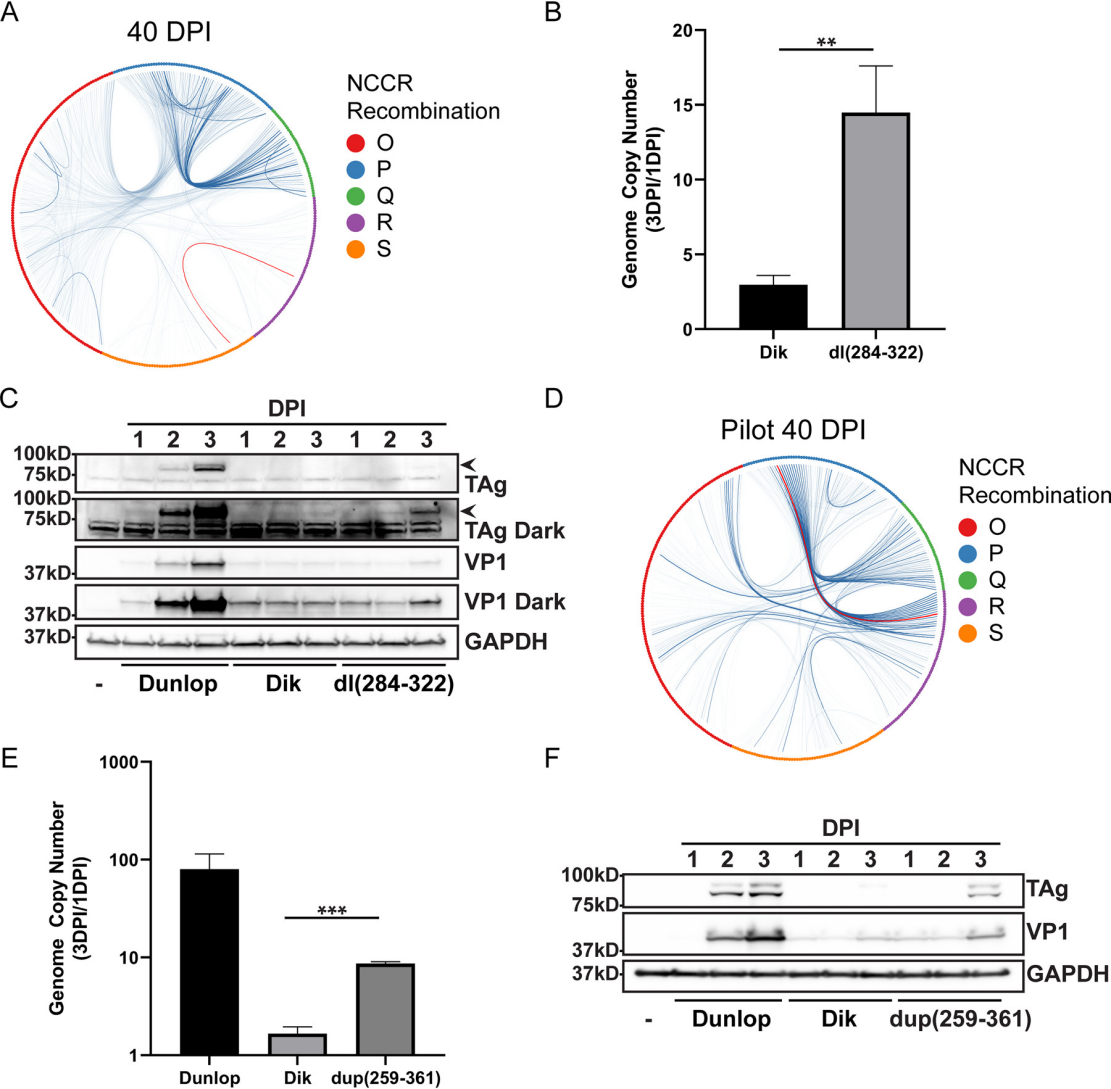
Genome replication and viral protein expression of wild-type and recombinant BKPyV. (A) BKPyV recombination junctions within the NCCR at 40 DPI are depicted as circular diagrams in which the junctions are linked with lines. The intensity of each line reflects the number of reads corresponding to that junction. The red event line represents the deletion in Dik dl(284–322). (B) Virus genome replication. Cells were infected with BKPyV wild type (Dik) and the recombinant Dik dl(284–322), and genome copy numbers were measured at 1 and 3 days postinfection (DPI) and normalized to mitochondrial DNA (mean ± SD; **, *P* ≤ 0.01). (C) Viral protein expression. Protein samples from infection with BKPyV Dik, Dik dl(284–322), or Dunlop were assayed for TAg (arrowhead) and VP1 expression. (D) NCCR circular diagram at 40 DPI. The red event line represents dup(259–361). (E) Virus genome replication. Cells were infected with BKPyV Dunlop, Dik, or Dik dup(259–361), and genome copy number increases were measured at 1 and 3 DPI and normalized to mitochondrial DNA (mean ± SD; ***, *P* ≤ 0.001). (F) Viral protein expression. Protein samples from the three viruses were assayed as in panel C.

To address the relationship between recombination and viral genome replication, we identified an abundant recombinant NCCR sequence and studied its effect on viral replication. To do this, we took an independent DNA sample that had been isolated at 40 days postinfection and amplified the NCCR using PCR. The products were separated on a nondenaturing polyacrylamide gel, and the two most abundant bands were isolated. After sequencing the PCR products, we determined that one band represented the starting archetype NCCR but the other contained a 39-bp deletion in the R and S blocks of the NCCR [Fig. 1B, Dik dl(284–322); Fig. 4A, red line]. According to our deep sequencing data, this deletion constitutes 3.45% percent of all NCCR recombinant reads at 40 days postinfection. To test the effect of this deletion on replication, we substituted it for the wild-type NCCR of the archetype genome. After growing this virus in 293TT cells, which complement the replication defect of archetype virus (22), RPTE cells were infected with both archetype virus and the archetype virus with the deletion [Dik dl(284–322)]. We harvested low-molecular-weight DNA and assayed DNA replication by qPCR. The results show a significant increase in replication in the Dik dl(284–322) virus compared to archetype virus (Fig. 4B). To examine viral protein expression, RPTE cells were infected with a rearranged variant (Dunlop), archetype virus (Dik), and the Dik dl(284–322) virus. Western blotting shows that the rearranged variant robustly expresses TAg and VP1 proteins, while the archetype virus does not express a detectable level of either protein (Fig. 4C). Dik dl(284–322) expresses both proteins at an intermediate level, consistent with the DNA replication data. This result indicates that recombination during persistent infection generates NCCR sequences that allow enhanced BKPyV protein expression and replication.

### Long-read single-molecule sequencing reveals complex and lethal recombination events.

Since full-length complete BKPyV genomes could not be assembled from the short-read sequencing results, we turned to long-read sequencing to examine complete recombinant genomes. Low-molecular-weight viral DNA from infected RPTE-hTERT cells during the exponential replication phase in the pilot experiment (Fig. 3A, right column) was amplified with rolling circle amplification (RCA). The RCA products were digested with BamHI to release linear BKPyV genomes, and the sequences of these genomes were read using the Nanopore MinION system. We did not detect any significant evidence of genomes that were not monomeric. The reads were aligned against an archetype genome template using BLAST. The results confirmed our observation that BKPyV accumulates extensive recombination events when replication begins. To characterize an individual genome, we cloned a dominant RCA product into pGEM7. This genome has a duplication of the P and Q blocks and a partial duplication of the R block [Dik dup(259–361)] (Fig. 1B; Fig. 4D, red line) but no other mutations within the rest of the genome. Our sequencing results showed that the dup(259–361) first appeared at 15 days postinfection. We propagated the Dik dup(259–361) virus in 293TT cells and examined its replication ability as we did for the Dik dl(284–322) variant above. The DNA replication result showed that there is a 9-fold increase in replication in the Dik dup(259–361) virus compared to the archetype virus (Fig. 4E). Western blotting showed that Dik dup(259–361) also expresses TAg and VP1 proteins at an intermediate level between the archetype virus and rearranged Dunlop variant (Fig. 4F). These results confirmed that recombination during persistent infection can lead to selection for viral genomes with enhanced replication ability compared to the archetype virus.

Our single-molecule results also demonstrated that multiple recombination events can be identified in a single BKPyV genome, with some of these events deleting and/or duplicating parts or all of viral protein-coding regions. Consistent with this finding, we isolated many recombinant genomes that were not capable of producing progeny in 293TT cells: we could propagate these genomes only as mixed populations (data not shown). In addition, we found a combination of the duplication in Dik dup(259–361) and additional recombination events in single viral genomes.

### Analysis of recombination junctions.

**FIG 5:**
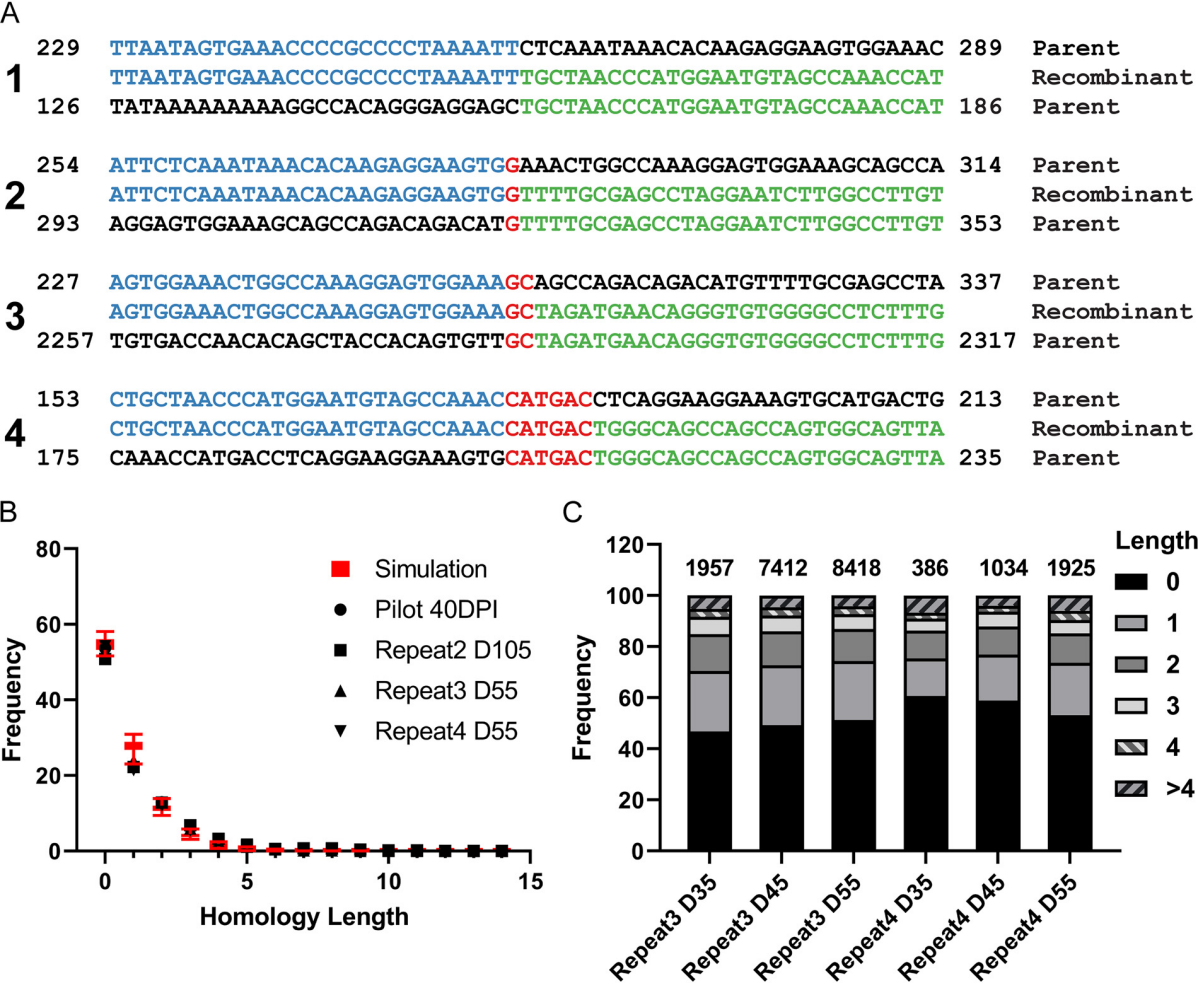
End joining analysis. (A) Representative recombination joints. Blue and green bases indicate the parental DNA fragments, and red bases indicate homology at the joints. Example 1 is Dik dup(259–361), and 2 is Dik dl(284–322). (B) Frequency of homology lengths at recombination joints. The lengths of homology at each unique recombination site were analyzed, and the frequency of each length was plotted (black symbols). The red box plots indicate the simulation as described in the text. (C) Distribution of homology lengths over time. The frequency of lengths at different time points from two representative repeats is shown. The numbers above the bars indicate the number of recombination events analyzed for each bar.

We next examined the sequences at the recombination junctions to determine if they might provide an indication of the recombination mechanism. It is extremely difficult to imagine that classical homologous recombination could lead to the types of duplications and deletions that are seen in NCCRs isolated from patients, as well as those that we detected. We therefore assumed that nonhomologous end joining (NHEJ) was being used to repair double-strand breaks that are known to occur during polyomavirus DNA replication (23). There are two types of NHEJ, classical (cNHEJ) and microhomology-mediated (MMEJ), both of which are error prone and can generate deletions and duplications (24). Instead of directly ligating DNA breaks together during cNHEJ, MMEJ takes advantage of a microhomology of less than 25 bp in size between the two parental DNA strands (25). Examples of recombination joints, including those of Dik dl(284–322) and Dik dup(259–361), are shown in Fig. 5A. We also graphed the distribution of lengths of homologous sequences at the joints to attempt to distinguish between cNHEJ and MMEJ (Fig. 5B). It is noteworthy that such homologous sequences could form from either cNHEJ or MMEJ. To attempt to distinguish the roles of cNHEJ and MMEJ, we simulated the distribution of homology lengths that would be generated by ligation of random ends using cNHEJ by computationally fragmenting the BKPyV genome and joining random fragments (Fig. S7). The range of homology lengths from 50 simulated random annealing experiments is illustrated as red whisker boxes in Fig. 5B, and our experimental data match the simulated results. However, MMEJ would yield a similar distribution unless one assumes that higher stretches of homology would be favored during the MMEJ process, which would have resulted in a nonlinear decay curve. Finally, we examined the distribution of homology lengths at the junctions over time. The results indicate that the relative frequency of lengths remains constant over the course of the infection (Fig. 5C).

10.1128/mBio.02356-21.7 FIG S7 Homology length simulation. See Materials and Methods for details. Download FIG S7, TIF file, 2.8 MB. Copyright © 2021 Zhao and Imperiale. 2021 Zhao and Imperiale. https://creativecommons.org/licenses/by/4.0/ This content is distributed under the terms of the Creative Commons Attribution 4.0 International license.

## DISCUSSION

BKPyV establishes a lifelong persistent infection in greater than 80% of the human population after initial exposure during early childhood (4); however, no practical model with which to study BKPyV persistence is currently available. A squirrel monkey model of BKPyV was reported in 2005 (26), but no subsequent studies adopted this model, likely because of the expense of research using nonhuman primates. Most *in vitro* models developed for BKPyV have focused on acute lytic infection. Our lab previously reported that RPTE cells could be applied to these studies for rearranged variants of BKPyV (13), and various labs now employ this model to study aspects of viral biology including the viral life cycle, the innate immune response, and the DNA damage response (27–35). However, RPTE cells cannot be used routinely for persistence studies because these primary cells start to grow slowly at passage 7 and stop replicating by passage 10, which limits the capability to use this primary cell line to study persistence for prolonged periods.

To establish a robust *in vitro* model of BKPyV persistence, we therefore extended the life span of RPTE cells by introducing hTERT to establish the RPTE-hTERT cell line (16). Our results show that archetype BKPyV genome copy number remains stable in these cells for up to 100 days before exponential genome amplification starts. Thus, archetype BKPyV successfully established a persistent infection in these cells. Because we harvested and diluted the cells every 5 days, the BKPyV genome must replicate at a steady low level to maintain a stable copy number. On the other hand, infection of RPTE-hTERT cells with an equal inoculum of rearranged virus results in death by day 5 (data not shown). Western blotting showed that viral TAg expression is not detectable during the persistent stage (Fig. 2C), but we cannot rule out that low-level TAg is required for genome maintenance. It is possible that the outcome in infection differs from cell to cell, as had been recently reported (36). However, we think that it is difficult to explain the varying length of persistence if this were the case. Persistence does not seem to be an artifact of hTERT expression since we were able to detect it in primary cells.

As the infection progressed in RPTE-hTERT cells, we detected an eventual exponential increase in BKPyV genome copy number that led to visible CPE and cell death. The time at which this occurred varied from repeat to repeat, which is consistent with a model in which recombination occurs randomly and leads to a replication-competent rearrangement(s) at different times in each repeat. Our deep sequencing and single-molecule sequencing results confirmed that the population of recombinants differs in each repeat. We also detected viral early and late protein expression when the genome amplified but not during the persistent phase. In addition, Western blotting indicated variations in the size of the TAg protein after genome amplification began, which suggests recombination occurred in the TAg gene. Indeed, this recombination within the early region was detectable in both the short-read and whole-genome sequences and may lead to changes in the length of the open reading frame or cryptic splicing of mRNA transcripts.

The deep sequencing results showing a high level of genome recombination occurring during persistent infection are consistent with clinical observations that variation exists in polyomaviruses isolated from patients with polyomavirus-related disease (11, 37–39). We detected new recombination events as persistent infection progressed, with most of the recombination in the NCCR (see Fig. S5 and S6 in the supplemental material). This indicates that recombination in the NCCR provides an advantage for viral genome replication and outgrowth, consistent with previous observations that the NCCR is the primary determinant of BKPyV replication (7, 11). We also observed genome recombination outside the NCCR, including some viral-host genome recombination. The recombination outside the NCCR seems to be less abundant, but this is likely because there is no selection for these events compared to events within the NCCR. Nonetheless, recombination was random throughout the genome and we did not detect a specific recombination site that is statistically different from the others in the terms of recombination frequency.

At this time, we do not know the mechanism by which the deletion and duplication in dl(284–322) and dup(259–361), respectively, enhance replication. While there are many putative transcription factor binding sites in the NCCR, only Sp1 has been found to actually bind and to play a significant role in the ability of BKPyV to replicate (12, 21). It will be interesting to determine if the recombination events we detected affect transcription, DNA replication, or both.

It is known that BKPyV infection induces a host cell DNA damage response (28, 40). In addition, interfering with the DNA damage response proteins dramatically impairs the quality and quantity of replicated viral genomes, suggesting that BKPyV replication is damage prone and continuously requires the DNA damage response pathway to resolve replication stress (28). Analysis of replicating viral DNA during infection with the simian polyomavirus SV40 has confirmed that the viral genome is subjected to single- and double-strand breaks. Under ataxia telangiectasia-mutated (ATM) inhibitor treatment, SV40 accumulates strand invasion and rolling circle replication intermediates, while with ATM- and Rad3-related kinase (ATR) knockdown, an increase in broken replication forks can be detected (23). To repair replication stress-associated DNA damage, cells have evolved mechanisms using both homologous recombination and NHEJ. A recent paper described a theoretical model for deletion and duplication in the NCCR (15); however, it cannot fully explain the extensive recombination we detect in the early and late regions of the BKPyV genome. In addition, the NCCR duplications and deletions that have been observed in patient isolates are difficult to explain if homologous recombination were the repair mechanism, and our results indicate that NHEJ, which is more likely to result in small deletions and duplications, is indeed the predominant mechanism.

It is thought that in patients, once rearrangement of the NCCR takes place, the rearranged virus will dominate the population and replace the archetype variant during viral replication. To confirm that rearrangement in the NCCR leads to activation of replication, we isolated a dominant NCCR by PCR amplification of DNA samples harvested after reactivation and used it to replace the archetype NCCR, as well as isolating a virus containing a rearranged NCCR by analysis of complete genomes using single-molecule DNA sequencing. Our results showed that a small deletion in the R and S blocks, or a duplication of P, Q, and part of R, leads to a robust increase in viral genome replication, which supports the model that recombination in the NCCR is sufficient to cause genome replication and viral protein expression. This is consistent with our previous finding that the NCCR is the major determinant of the differential replication ability of archetype and rearranged viruses (7). It is also consistent with the observation that many types of NCCR deletions and/or duplications can all lead to enhanced replication (9, 11, 12). It will be interesting to determine how these rearrangements affect transcription of viral genes and the efficiency of DNA replication initiation at the origin.

We also isolated recombined genomes that have sizeable deletions in virus protein-coding sequences. Subsequent experiments showed that these viruses cannot replicate independently, as would be expected: they could be passaged as mixed populations of genomes but did not grow out upon limiting dilution of the viral lysates. However, the fact that these sequences are amplified in the cell suggests that some recombinant genomes may function as helper viruses, with these defective genomes behaving like helper-dependent viruses. This is consistent with clinical findings in which recombinant BKPyV genomes that carry protein-coding sequence deletions can be isolated from urine and kidney allograft biopsy specimens from kidney transplant recipients. These isolated viruses were naturally defective in producing infectious progeny; however, complementation in *trans* with the missing viral protein could rescue this defect (37).

In this study, we developed a powerful *in vitro* persistence and reactivation model for BKPyV. We also developed a bioinformatics pipeline for analyzing circular viral DNA recombination. Our model demonstrates that the BKPyV can evolve fairly rapidly, and some of the rearrangements in the NCCR eventually trigger enhanced viral protein expression and DNA replication and lead to obvious CPE and production of viable viral progeny. Our model mimics an accelerated recombination process in the kidneys of renal transplant patients without an immune system and suggests that BKPyV recombination and enhanced replication are a coordinated process that leads to disease. Our study is the first to demonstrate that recombination can occur throughout the viral genome: we propose that most of these recombination events are negatively selected due to their lethality and therefore were not detectable in previous studies examining patient isolates. Our results indicate that BKPyV, even though it is a DNA virus with a low DNA polymerase error rate, can evolve rapidly within an individual host due to recombination. Our model can be used to further study the evolution and reactivation of BKPyV in the kidney, including identification of viral and host factors that contribute to these processes.

## MATERIALS AND METHODS

### Cell culture.

RPTE and RPTE-hTERT cells were maintained in REGM BulletKit media (REGM/REBM; Lonza; CC-3190) at 37°C with 5% CO_2_ in a humidified incubator (16). Cells were passaged by detaching with 0.25% trypsin-EDTA and split at 1:4 for maintenance.

### BKPyV purification and infection.

Plasmids containing the viral genome were digested with BamHI to excise the genome from the vector and then recircularized with T4 ligase. The recircularized genome was transfected into 293TT cells with PEI Max transfection reagent (Polysciences; 24765-1). Progeny viral particles were purified on a linear cesium chloride gradient, and titers were determined as described previously (27, 41). For infection, cells were prechilled for 15 min at 4°C. One milliliter of the virus inoculum was added to one well of a 6-well plate to give a multiplicity of infection (MOI) of 5,000 genomes/cell and incubated at 4°C for 1 h with gentle shaking every 15 min to distribute the inoculum over the entire well. The plate was transferred to 37°C after the 1-h incubation. As we cannot determine the titer of archetype virus using a standard infectious unit (IU) assay due to its inability to replicate, we use genome numbers to normalize different viruses. For reference, 5,000 genomes of a rearranged variant (Dunlop) equal approximately 5 IU.

### Low-molecular-weight DNA isolation.

Low-molecular-weight DNA was harvested with a modified Hirt extraction protocol developed by the Buck laboratory (42). Briefly, cells were suspended in 250 μl buffer I (50 mM Tris, pH 7.5; 10 mM EDTA; 50 μg/ml RNase A; 20 U/ml RNase T_1_ cocktail) and then lysed by adding 250 μl buffer II (1.2% SDS) and incubating for 5 min at room temperature. Cellular DNA was precipitated by adding 350 μl buffer III (3 M CsCl; 1 M potassium acetate; 0.67 M acetic acid), incubating at room temperature for 10 min, and centrifuging at 16,000 × *g* for 10 min. The supernatant was transferred to minispin DNA purification columns (Epoch Life Science). Five hundred microliters PB and 750 μl PE buffer (Qiagen) were added sequentially to the column and centrifuged to wash the DNA. Low-molecular-weight DNA was eluted with 50 μl EB buffer (Qiagen).

### Quantitative PCR.

DNA samples were first diluted with DNA-grade water at 1:200. Primer pairs amplifying large tumor antigen (TAg) and mitochondrial 16S rRNA gene segments were used as previously described (43). The Shapiro-Wilk test, F-test, and Student *t* test were performed with GraphPad Prism.

### Western blotting.

Protein samples were harvested with E1A buffer, electrophoresed, transferred, and probed as previously reported (16).

### Short-read sequencing.

The concentration of low-molecular-weight DNA was measured with a PicoGreen double-stranded DNA (dsDNA) assay kit (Invitrogen). Sequencing libraries were prepared and pooled according to the manual of the transposon-based Illumina DNA Prep kit (Illumina). Paired sequences of 251 bp were read using the Illumina MiSeq system with MiSeq reagent kits v2. This sequencing was performed at the University of Michigan Sequencing Core.

### Long-read sequencing.

Circular low-molecular-weight DNA was rolling circle amplified with the TempliPhi amplification kit (Cytiva). Amplified DNA was digested with BamHI to release linear single genome-length molecules. The digested DNA was separated on a 1% agarose gel, and the ∼5-kb genomic DNA band was harvested with a QIAquick gel extraction kit (Qiagen). The DNA library was prepared according to the manual for the ligation sequencing kit (Nanopore). Long-read DNA sequences were read with the Nanopore MinION system in our laboratory.

### Bioinformatic recombination analysis.

(Step 1) Discard viral DNA reads that do not contain a recombination event. Deep sequencing results (*.fastq.gz) were parsed, and each sequencing read (251 bp in length) that perfectly matches the BKPyV template was omitted from further analysis. (Step 2) Discard nonviral DNA reads. Remaining reads were broken into 10 25-mers. If none of the 10 25-mers matched BKPyV, the original read was omitted from further analysis. All reads that were not omitted were collected to a file in FASTA format for subsequent BLAST. (Step 3) BLAST. All sequences collected in the previous step were subjected to BLAST search against the archetype genome sequence with local BLAST+. (Step 4) Format BLAST results. Indexes of “start of alignment in query, end of alignment in query, start of alignment in subject, and end of alignment in subject” were parsed from the BLAST results. When a read comprised more than one stretch of DNA originating from different locations of the BKPyV Dik genome, it was considered a recombination event. In addition, the “aligned segment of the query sequence” and “aligned segment of subject the sequence” were parsed from BLAST results, and recombination joints were determined by assembling these two aligned segments as illustrated in Fig. 5A. (Step 5) Generate recombination diagrams. An R script was used to draw the circular diagrams. Recombination histograms were drawn with Prism.

### Homology length analysis and simulation.

After formatting the BLAST result (step 4, bioinformatic recombination analysis), the homology lengths at the recombination joints were analyzed based on sequence overlap. To simulate homology formation by NHEJ, the BKPyV sequence was first computationally broken into a set of 26-mers, each differing at their 5′ end by 1 bp. Random unique 26-mers were religated, and the homology length at the center was recorded. If no homology was present at the center, the homology length was recorded as 0. Simulations were performed 50 times with 1,000 random annealing events each. Minimum to maximum values are shown with whisker boxes.

### Construction of viral genome with NCCR deletion.

An NCCR containing a deletion of nucleotides 284 to 322 was synthesized with SpeI and SacII sites at the ends and used to replace the archetype NCCR in pGEM-Dik3site (7). The entire genome was confirmed by DNA sequencing. Virus was propagated as previously described, and its sequence was verified again before use in experiments (22).

### Recombinant viral genome isolation.

Gel-purified DNA from rolling circle amplification was cloned into pGEM-7Zf(+)at the BamHI site using T4 ligase. Ligation products were transformed into XL10 cells (Agilent). Colonies containing recombinant genomes were isolated, and DNA was prepared and sequenced. Virus was propagated as previously described, and its sequence was verified again before use in experiments (22).

### Data availability.

All sequences have been posted to NCBI under accession number PRJNA753127. Code used in this report can be found at https://github.com/LBZhao/BK_Polyomavirus_recombination_analysis.
