# Supplementary figures and images for: A Cell Culture Model of BK Polyomavirus Persistence, Genome Recombination, and Reactivation
Source: mBio. 2021 Sep 2;12(5):e02356-21. doi: 10.1128/mBio.02356-21 (PMC8546605; doi:10.1128/mBio.02356-21)

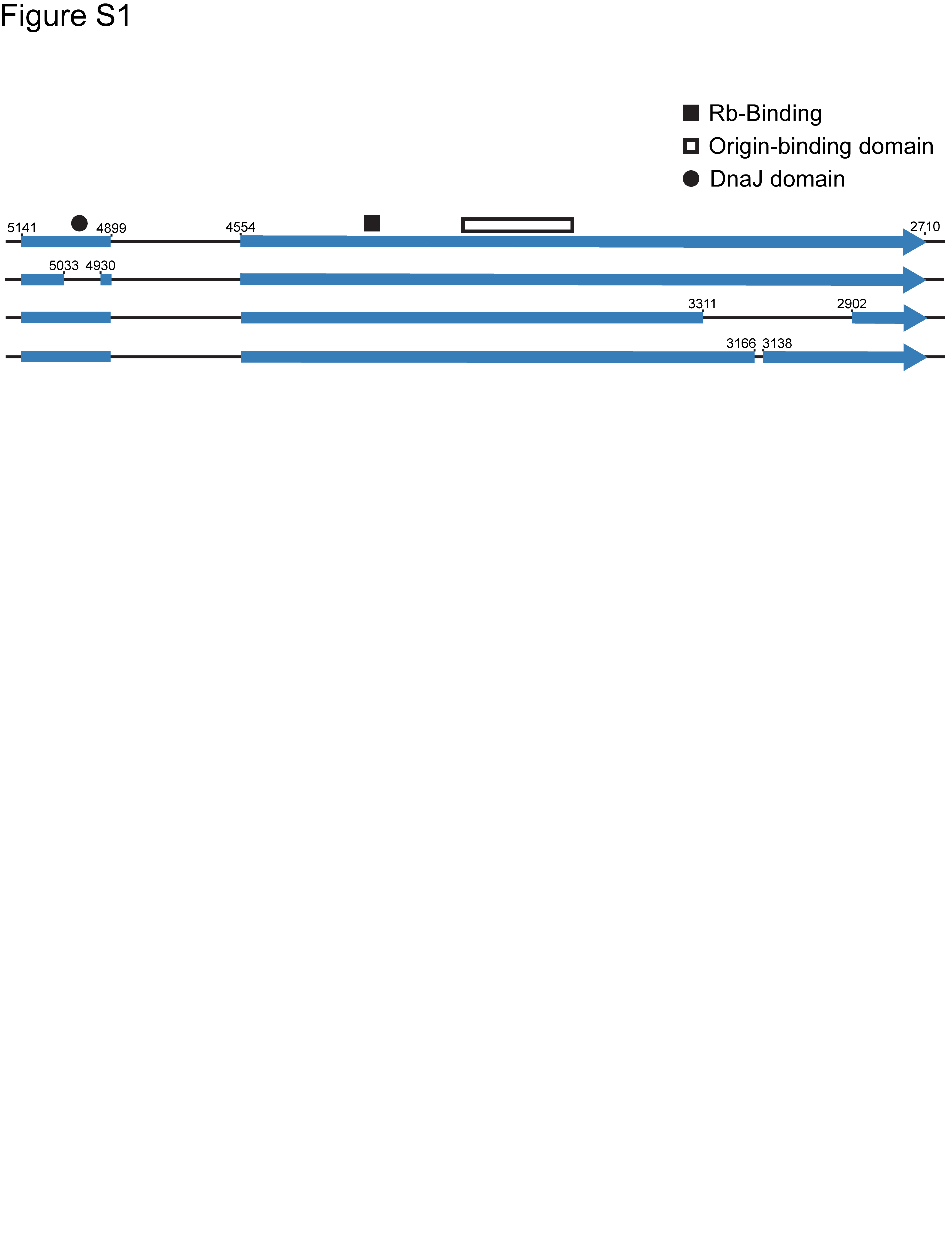

Supplement: FIG S1 [file mbio.02356-21-sf001.tif]

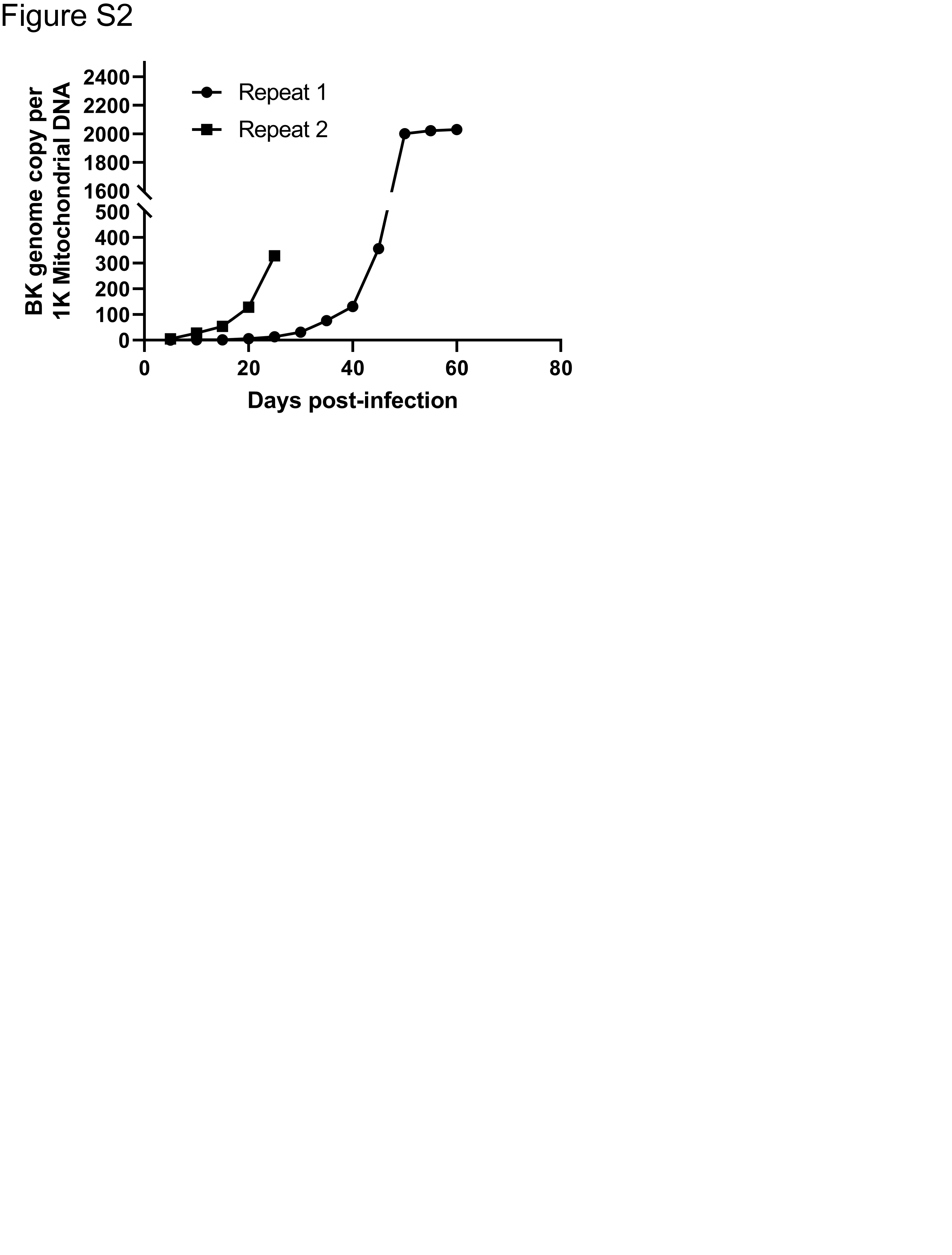

Supplement: FIG S2 [file mbio.02356-21-sf002.tif]

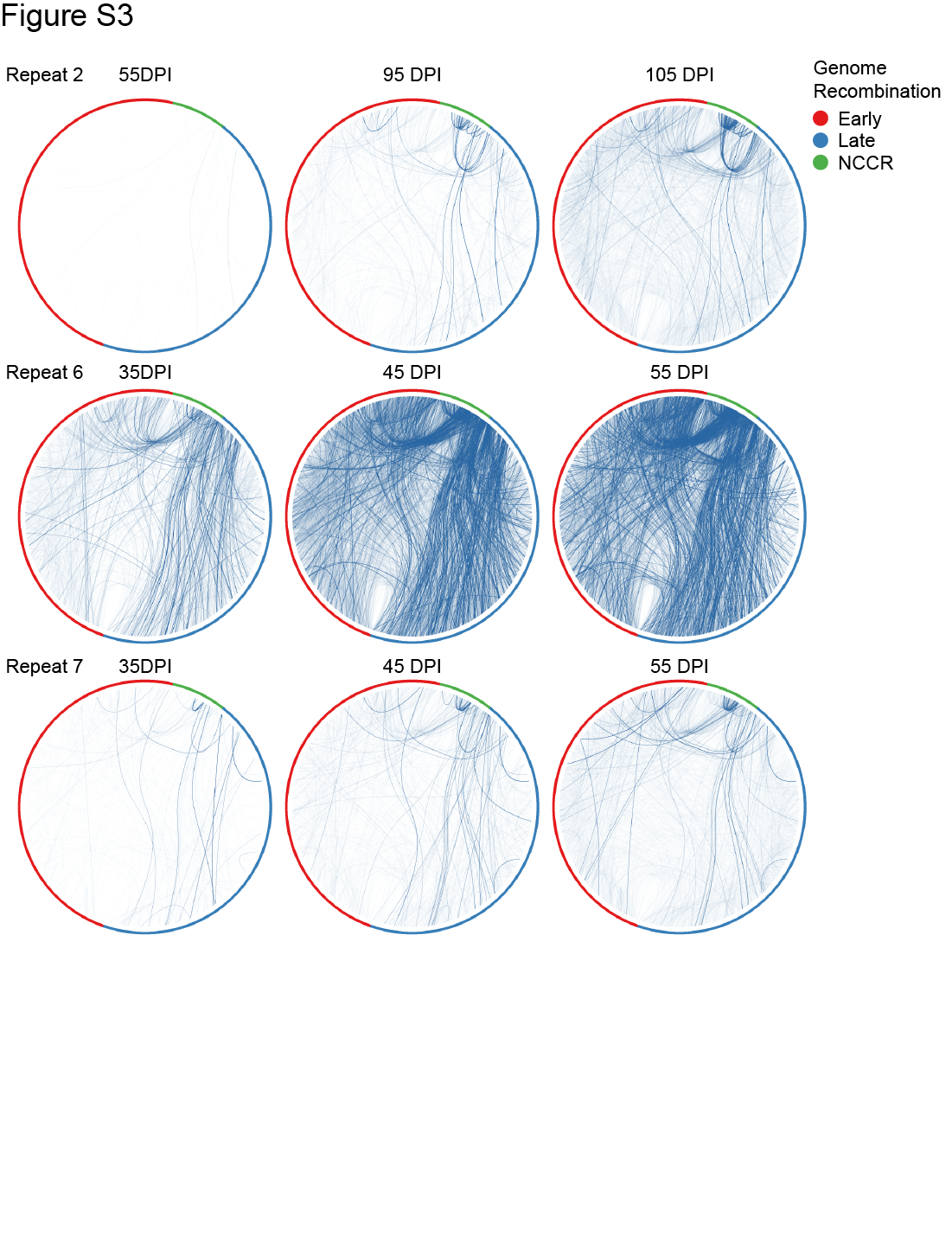

Supplement: FIG S3 [file mbio.02356-21-sf003.tif]

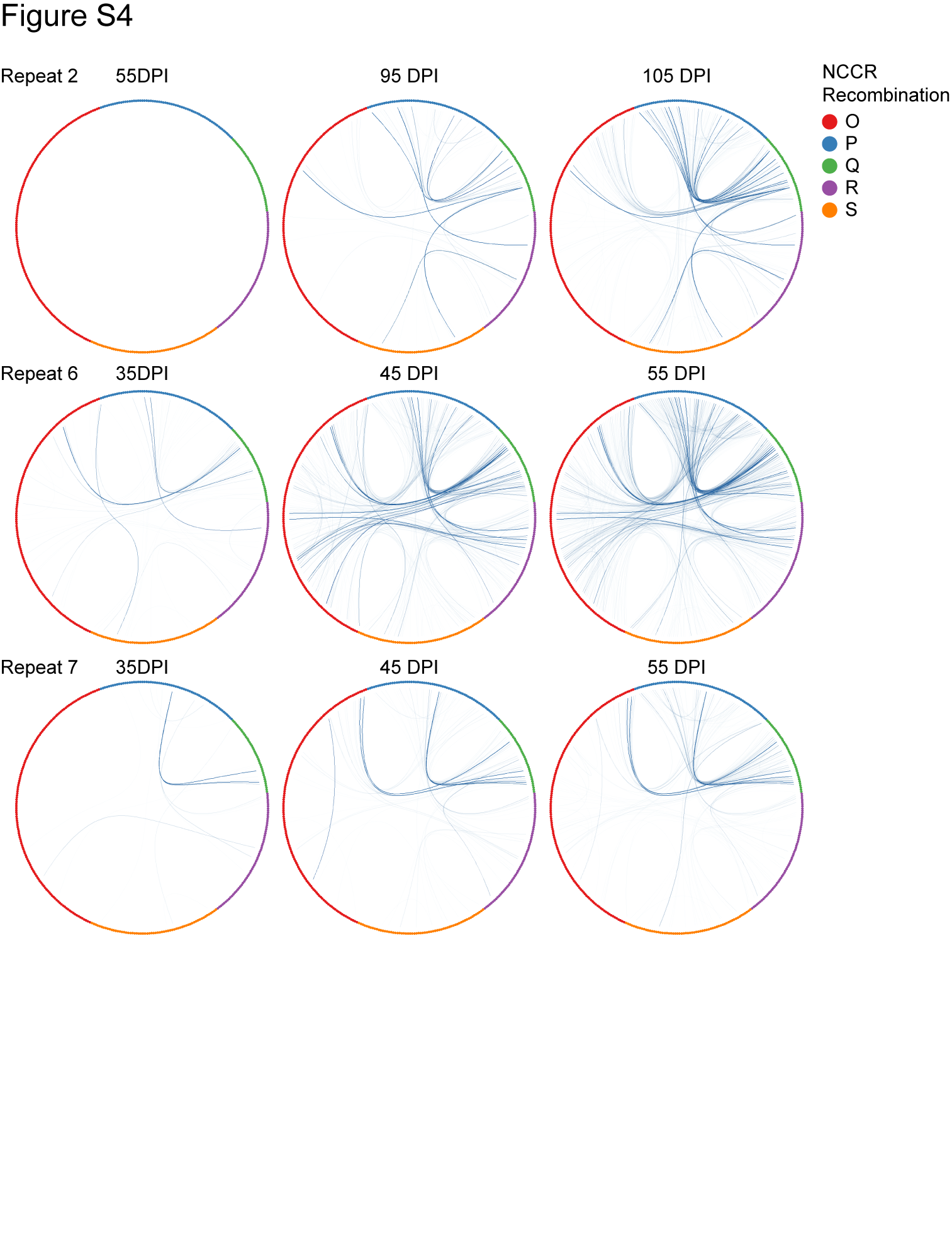

Supplement: FIG S4 [file mbio.02356-21-sf004.tif]

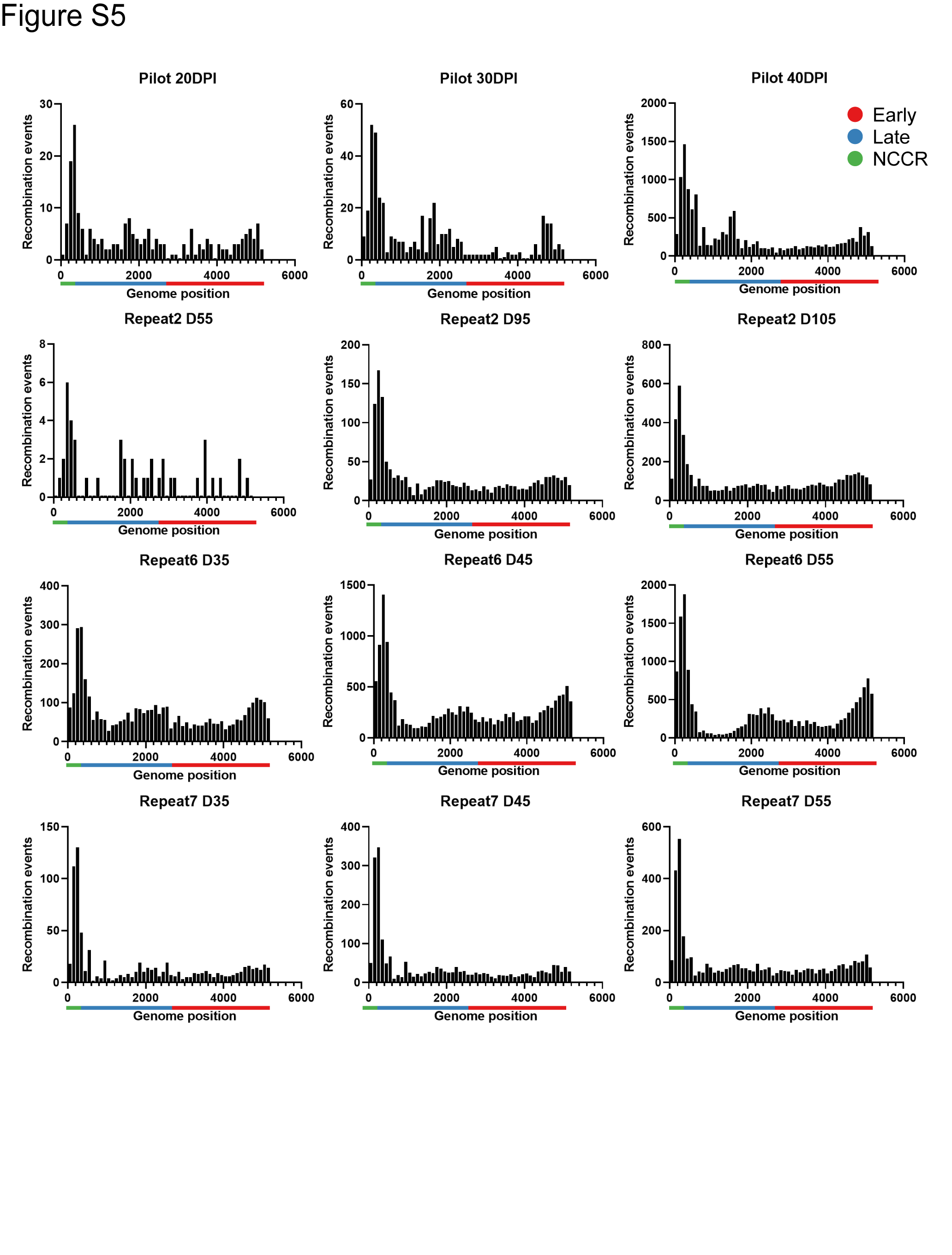

Supplement: FIG S5 [file mbio.02356-21-sf005.tif]

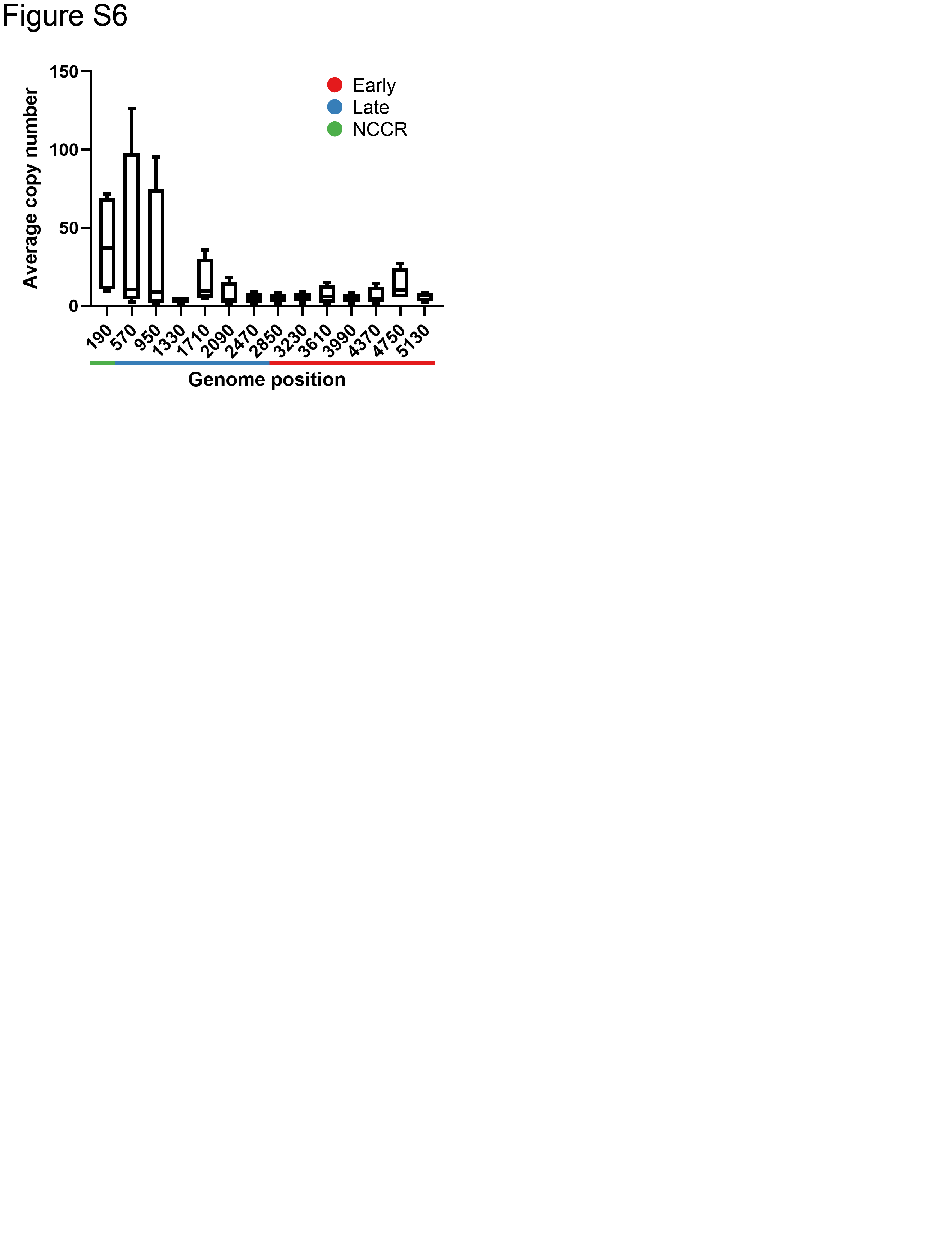

Supplement: FIG S6 [file mbio.02356-21-sf006.tif]

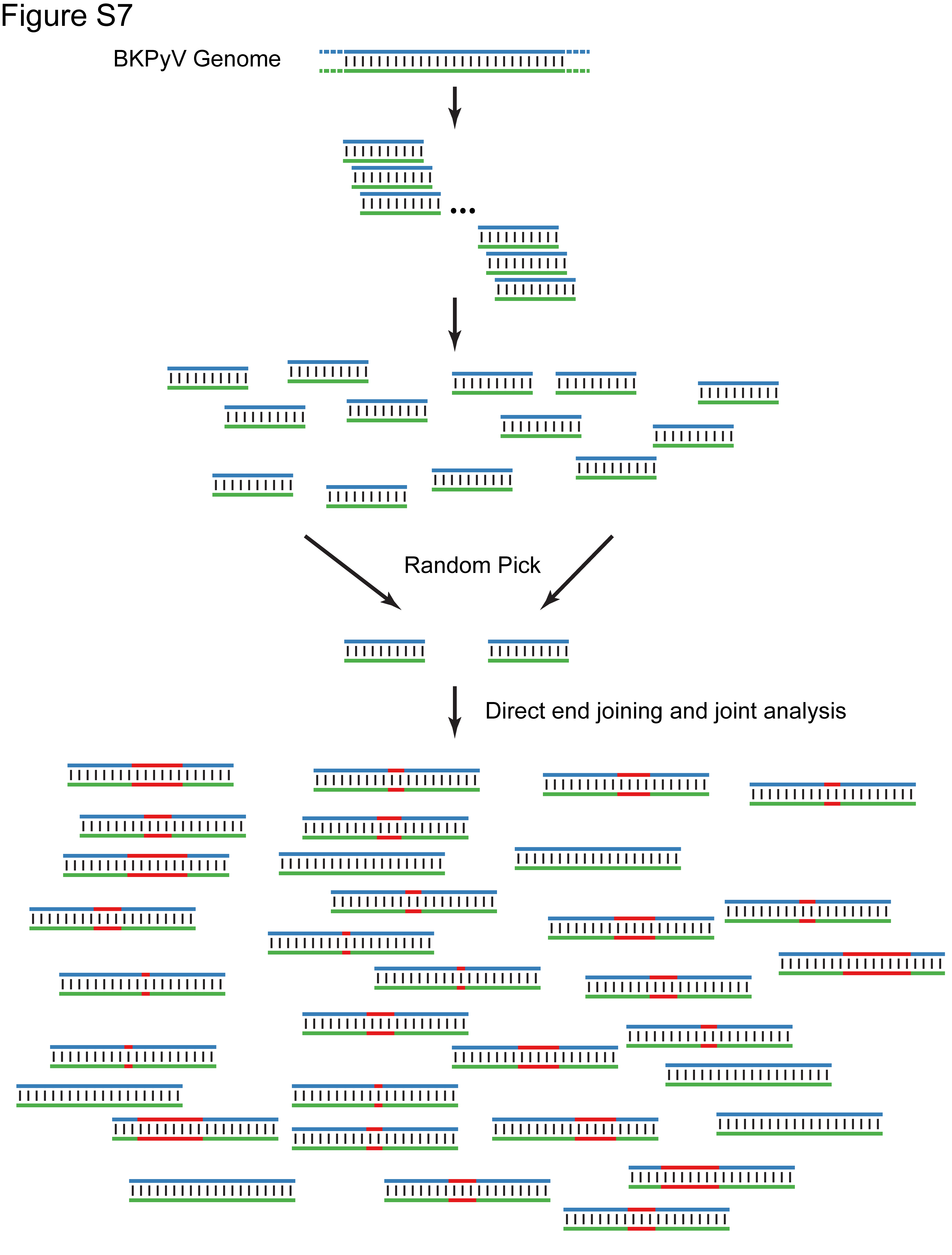

Supplement: FIG S7 [file mbio.02356-21-sf007.tif]
